# Non-kinase targeting of oncogenic c-Jun N-terminal kinase (JNK) signaling: the future of clinically viable cancer treatments

**DOI:** 10.1042/BST20220808

**Published:** 2022-12-01

**Authors:** Sharissa L. Latham, Yolande E.I. O'Donnell, David R. Croucher

**Affiliations:** 1 The Kinghorn Cancer Centre, Garvan Institute of Medical Research, Sydney, Australia; 2 St Vincent's Hospital Clinical School, UNSW, Sydney, Australia

**Keywords:** breast cancers, cancer, drug discovery and design, JNK, molecular scaffolds, therapeutics

## Abstract

c-Jun N-terminal Kinases (JNKs) have been identified as key disease drivers in a number of pathophysiological settings and central oncogenic signaling nodes in various cancers. Their roles in driving primary tumor growth, positively regulating cancer stem cell populations, promoting invasion and facilitating metastatic outgrowth have led JNKs to be considered attractive targets for anti-cancer therapies. However, the homeostatic, apoptotic and tumor-suppressive activities of JNK proteins limit the use of direct JNK inhibitors in a clinical setting. In this review, we will provide an overview of the different JNK targeting strategies developed to date, which include various ATP-competitive, non-kinase and substrate-competitive inhibitors. We aim to summarize their distinct mechanisms of action, review some of the insights they have provided regarding JNK-targeting in cancer, and outline the limitations as well as challenges of all strategies that target JNKs directly. Furthermore, we will highlight alternate drug targets within JNK signaling complexes, including recently identified scaffold proteins, and discuss how these findings may open up novel therapeutic options for targeting discrete oncogenic JNK signaling complexes in specific cancer settings.

## Introduction

The c-Jun N-terminal Kinases (JNKs) are members of the mitogen-activated protein kinase (MAPK) family that critically regulate a diverse and somewhat opposing range of physiological processes, including cell death, proliferation, differentiation and invasion [1]. This functional diversity is achieved through the assembly of spatially and compositionally discrete multi-protein complexes, which integrate and transmit signals in response to various stimuli. Although originally named stress-activated protein kinases (SAPKs) for their profound response to extracellular stress stimuli such as UV-irradiation, heat shock, osmotic stress, reactive oxygen species and inflammatory cytokines, it is now apparent that JNKs are also activated by intracellular stimuli and biomechanical cues, and that persistent JNK activation underlies pathogenesis in a number of disease contexts, including cancer [2,3].

**Figure 1.**
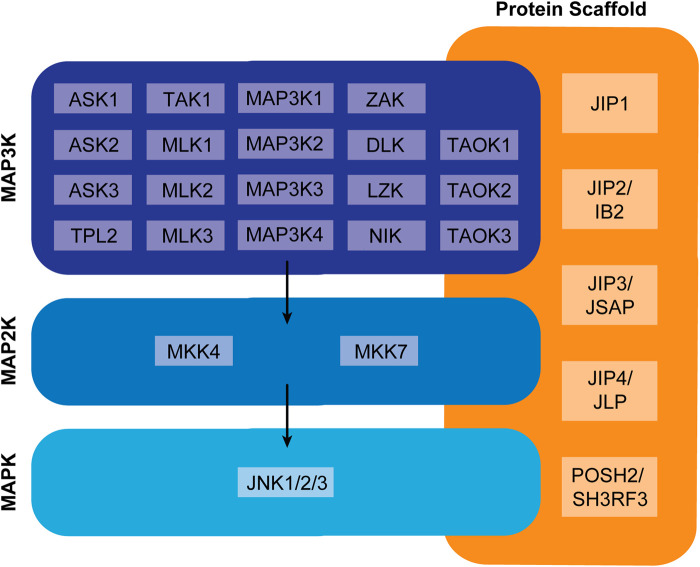
Complexity of JNK signaling. JNK proteins are activated downstream of a diverse range of upstream MAP2Ks and MAP3Ks. The precise MAP3K-MAP2K-MAPK composition of a given signaling complex is influenced by tissue and cell type, physiological/pathological context, and scaffold proteins, which bind more than two signaling components and direct them to discrete subcellular locations.

JNK signaling conforms to the hierarchal MAPK network structure, whereby one of several MAPK-kinase-kinases (MAP3Ks) phosphorylates and activates one of two MAPK-kinases (MAP2K), MKK4 or MKK7, which in turn activate the JNK isoforms, JNK1, JNK2 and/or JNK3 (Figure 1). Each of these JNK isoforms are subject to alternative splicing, with JNK1 and JNK2 variants expressed ubiquitously and JNK3 variants restricted to the brain, heart and testis. Whilst this implies that the composition of JNK signaling complexes is inherently tissue and cell type-dependent, a significant body of research now demonstrates that scaffold proteins also play a key role in dictating MAP3K–MAP2K–MAPK combinations and complex localization [4,5]. By restricting JNK activation to discrete subcellular compartments, scaffold proteins limit JNKs access to substrates such as transcription factors, translation machinery, hormone receptors, apoptotic effectors and cytoskeletal proteins. Once active, JNKs phosphorylate serine/threonine-proline motifs within these spatially segregated substrates in order to drive context specific biological responses [6].

Given the diversity of these JNK-regulated processes, JNK signaling has been implicated in a number of pathophysiological conditions, including neuro-degenerative diseases, diabetes and cancer. In the context of cancer, JNK hyper-activation has been reported in multiple solid tumor types and hematological malignancies, with countless studies demonstrating the therapeutic potential of JNK-targeting strategies [3]. However, a significant body of evidence has also elucidated key tumor-suppressive roles for JNK [7]. This dichotomy of JNK functions is particularly evident in the context of breast tissue, where *in vivo* JNK1/JNK2 knockout models have demonstrated that JNK plays essential roles in maintaining the architecture of normal breast tissue [8], driving the genetic programs required for mammary gland involution post lactation [9] and preventing early tumor initiation events [10,11]. These tumor-suppressive roles starkly contrast JNKs tumor-promoting functions in breast cancer tissue, which includes driving primary tumor growth, promoting an immunosuppressive tumor microenvironment, positively regulating cancer-stem cell populations, promoting tumor cell migration and invasion, and modulating both the structure and immunological landscape of the metastatic niche to support metastatic disease progression [12–15]. Furthermore, an extra layer of complexity is added by the critical requirement for JNK activity in the apoptotic response to chemotherapeutic intervention in breast cancer treatment [16].

Whilst there is significant interest in therapeutically targeting the pro-tumorigenic functions of JNK in various cancers, the pleotropic nature of JNK signaling means that direct JNK inhibition may have adverse consequences and is unlikely to yield clinically viable cancer treatments. This is reflected in the existing clinical trials that have already been performed with JNK inhibitors (Table 1), which were predominantly performed in the context of fibrotic or inflammatory diseases. A number of these studies are testing the long-term tolerability of JNK inhibitors, but not within an oncology setting and with no long term monitoring of potential neoplastic activity. With this in mind, we will provide an overview of the different JNK targeting strategies developed to date including various ATP-competitive and non-competitive inhibitors, and outline the insights that these compounds and peptides have provided regarding JNK-targeting in cancer along with their potential limitations. Furthermore, we will highlight recent studies describing novel JNK scaffolds that regulate cancer stemness, and discuss the implications that discoveries such as these may have on the design and development of JNK-targeting therapies in the future.

**Table 1:** JNK inhibitors assessed in clinical studies

Inhibitor type	Inhibitor name	Preclinical cancer models		Clinical trials					
		Effects	Refs	Condition/disease	Identifier	Phase	Treatment details	Outcome	Refs
ATP- competitive inhibitor	CC-401	- Inhibits TNBC primary tumor growth and metastasis - Sensitizes colon cancer to chemotherapy *in vivo*	[12,13,24]	High-risk myeloid leukemia	NCT00126893	I	No information available	Terminated; reason not cited; N/P	
	CC-930 Tanzisertib	- Not tested in cancer models		Healthy adults	Unknown	I	Three-way crossover study, daily oral administration for 6 days with 7 days washout	Completed; well tolerated with no serious adverse events reported	[40]
				Healthy males	Unknown	I	Single-dose of [^14^C]-Tanzisertib, oral	Completed; eliminated via urinary and fecal excretion with no unique metabolites	[96]
				Idiopathic pulmonary fibrosis	NCT01203943	II	Repeated oral administration for up to 56 weeks	Terminated; benefit/risk profile cited; adverse events in 46.4% of subjects	[40]
				Discoid lupus erythematsous	NCT01466725	II	Daily treatment for 4–8 weeks	Terminated; benefit/risk profile cited; N/P	
	CC-90001 BMS-986360	- Not tested in cancer models		Healthy adults	NCT02110420	I	Single and multiple ascending doses, oral	Completed; safe and well-tolerated	[97]
					NCT02321644	I	Multiple doses and single dose fed/fasting conditions, oral		
					NCT03958864	I	Multiple doses, oral		
					NCT03363815	I	Multiple doses, tested with the following combinations and fed/fasting conditions: + Ozeprazole, Midazolam, Warfarin, Vitamin K + Rosuvastatin + Metformin, Digoxin + Nintedanic	Completed; N/P	
				Healthy males	NCT04655898	I	Single-dose of [^14^C]-CC-90001, oral	Completed; N/P	
				Hepatic impairment	NCT03742882	I	Single-dose, oral	Completed; N/P	
				Pulmonary fibrosis	NCT02510937	Ib	Daily oral administration for 12 continuous weeks	Completed; N/P	
				Idiopathic pulmonary fibrosis	NCT03142191	II	Daily oral administration for 24-104 weeks	Completed; trial setup published; data N/P	[42]
				Non-alcoholic steatohepatitis and liver fibrosis	NCT04048876	II	Daily oral administration, time not specified	Terminated; changed business objectives cited; N/P	
	AS602801 PGL-5001 Bentamapimod	- Cytotoxic in human pancreatic, non-small cell lung, ovarian and gliobastoma cells - Sensitizes ovarian cancer stem cells to chemotherapy - Perturbs prostate cancer cell invasion *in vitro*, and tumor growth *in vivo*	[25–29]	Inflammatory endometriosis	NCT01630252	IIa	Daily oral administration for up to 5 months accompanied by 1–2 Depot Medoxyprogesterone Acetate (DMPA) injections	Completed; N/P	
Substrate-competitive inhibitor	D-JNKI1 XG-102 AM-111 Brimapitide	- Reduces HCC proliferation *in vitro* and tumor growth *in vivo* - Reduces cancer pain and tumor growth in murine skin cancer model	[55,56]	Healthy males	NCT01570205	I	Single-dose, intravenous	Completed; N/P	
				Acute sensorineural hearing loss	Unknown NCT00802425 NCT02561091 NCT02809118 EudraCT 2013-002077-21	I/II II III III III	Single-dose, intratympanic Single-dose, intratympanic Frequency and timing not specified; intratympanic Single-dose, intratympanic Single-dose, intratympanic	Completed; safe and well-tolerated Completed; N/P Completed; N/P Terminated; data available from another study cited; N/P Completed; Otoprotective	[58,59]
				Intraocular inflammation	EudraCT 2011-000171-14 (cited identifier invalid)	Ib	Single-dose, subconjunctival	Completed; Safe and well tolerated	[98]
				Post-cataract surgery intraocular inflammation	NCT02235272 NCT02508337	III III	Single-dose, subconjunctival Single-dose, subconjunctival	Completed; N/P Completed; N/P	

N/P, Data not published; NCT identifiers from ClinicalTrials.gov; EudraCT identifiers from eudract.ema.europa.eu.

## ATP-competitive JNK inhibitors

**Figure 2.**
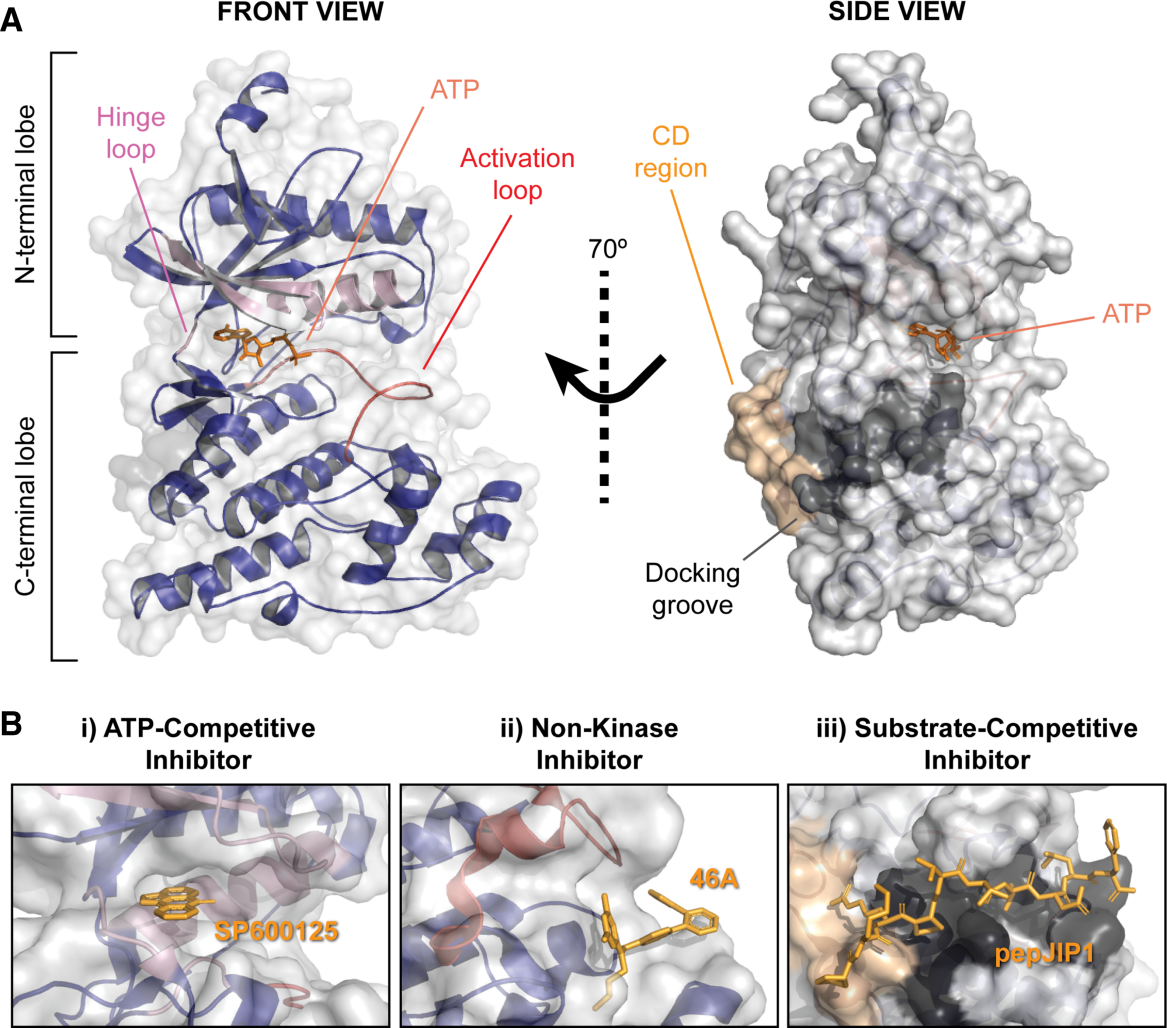
Structural features and inhibitor binding sites of JNKs. (**A**) JNK proteins are comprised of distinct N-terminal and C-terminal lobes that surround an inner ATP-binding cleft, where ATP (orange) docks. This ATP binding cleft consists of the hinge loop, G-Rich loop, C-helix (all in light pink) and the activation loop (red), which encompasses the TPY motif for dual MAP2K phosphorylation and activation. The docking site (D-site), the interface through which JNKs interact with most of their binding partners, is located next to the hinge loop and comprises both the CD region (beige) and hydrophobic docking groove (dark grey). This figure was assembled using the AlphaFold structure prediction for JNK1 (AF-P45983-F1) [94,95]. The ATP shown in the nucleotide binding cleft was obtained by overlaying the AlphaFold prediction with a structure of human JNK1 (PDB 2XRW; RMSD = 0.633 (Å)). (**B**) Three main types of JNK inhibitors have been described, including (i) ATP-competitive, (ii) non-kinase and (iii) substrate competitive JNK inhibitors. (i) ATP-competitive JNK inhibitors such as SP600125 work by displacing ATP from the nucleotide binding pocket (lPDB 1UKI). (ii) The two biaryl tetrazole non-kinase JNK inhibitors that have been identified, including 46A, bind to inactive JNKs and prevent their phosphorylation by upstream MAP2Ks (PDB 302M). (iii) Alternatively, substrate-competitive JNK inhibitors such as pepJIP1 interact with the D-site and effectively block JNKs interactions with its binding partners, including scaffold proteins, upstream MAP2Ks, downstream substrates and phosphatases (PDB 1UKI).

In terms of protein structure, JNKs comprise distinct N- and C-terminal lobes that are linked by a flexible hinge loop (Figure 2A). This hinge loop and the structural elements surrounding the central cavity between the two lobes form the ATP-binding cleft. ATP-competitive JNK inhibitors effectively block the functions of JNK proteins by competing with and replacing ATP at this site. Given that JNK dysregulation has been implicated in multiple disease contexts, there has been significant research into the development of these type I inhibitors. The structural characteristics of this extensive list of inhibitors and their precise binding mechanisms have been reviewed elsewhere [17,18].

ATP-competitive JNK inhibitors have been crucial for delineating and defining our understanding of JNK signaling in tumorigenesis, with the anthrapyrazole inhibitor SP600125 amongst the most commonly used in experimental cancer models [19] (Figure 2B-i). Whilst SP600125 has demonstrated significant anti-tumor potential in different cancer types [20–22], its lack of specificity for JNK has generated controversy [23], limited its use and driven the development of second-generation inhibitors, such as CC-401. Although there is limited peer-reviewed biochemical data available for CC-401, recent *in vivo* studies have demonstrated that this well-tolerated compound can sensitize colon cancers to various treatments [24], and inhibit metastatic triple negative breast cancers (TNBCs) by blocking JNK-dependent primary tumor growth, cancer stemness, invasion and metastatic niche development [12,13]. Despite these promising *in vivo* results, a Phase 1 clinical safety, pharmacokinetics and pharmacodynamics study of CC-401 conducted in patients with high-risk myeloid leukemia (NCT00126893; clinicaltrials.gov) was terminated for unknown reasons and the compound not pursued further in clinical settings. Another ATP-competitive JNK inhibitor, AS602801, has also shown promising results within pre-clinical cancer models [25–29] and proceeded to clinical trial for the treatment of inflammatory endometriosis (Table 1). However, the limited biochemical and clinical data available for this compound prevents any discussion of its potential uses and limitations.

Improved JNK specificity has also been achieved through the development of irreversible JNK inhibitors, such as JNK-IN-8, which elicits sustained effects through the formation of a covalent bond with a cysteine in the ATP-binding cleft [30]. Although primarily used as an *in vitro* tool, JNK-IN-8 has been shown to sensitize both pancreatic ductal adenocarcinomas and TNBCs to various therapeutic agents *in vivo* [31,32], and inhibit TNBC primary tumor growth and lung metastasis by modulating the immunological landscape of the tumor microenvironment [15]. Whilst many articles refer to the harmful consequences of type I JNK inhibitors, there is limited literature detailing their side effects. However, it is within reason that these pan-JNK inhibitors may recapitulate the phenotypes observed in compound genetic knockout models, where the homeostatic, apoptotic and tumor-suppressive activities of JNK are adversely perturbed [8,11,33].

These limitations of pan-JNK inhibitors, along with the paradoxical roles of JNK isoforms in various physiological processes and disease settings, including cancer (reviewed in [7,34]), have prompted significant interest in the development of isoform selective inhibitors. Using rational drug design, a number of ATP-competitive inhibitors with enhanced selectivity for JNK3 have been described [35–37]. In terms of JNK1 and JNK2 selectivity, compounds such as the Celgene Corporation inhibitors CC-930 and CC-90001 have been shown to preference one isoform over another, with biases towards JNK2 and JNK1, respectively [38,39]. Whilst Phase 2 studies assessing CC-930 (also Tanzisertib) in patients with Discoid Lupus Erythematsous and Idiopathic Pulmonary Fibrosis were terminated due to the benefit/risk profile [40] (Table 1), CC-90001 passed Phase 1/1b trials with an acceptable safety profile and results are anticipated from a recently completed long-term Phase 2 study in patients with Idiopathic Pulmonary Fibrosis [41,42]. Pending safety results, it would be of great interest to evaluate CC-90001 in the context of hepatocellular carcinoma (HCC) and lung cancer [43–45], where JNK1, but not JNK2, has been implicated in tumor progression.

## Non-kinase JNK inhibitors

Outside of the ATP-binding pocket, two non-ATP binding sites have been identified for small-molecule and/or peptide-based JNK inhibitors. The first of these, reported by Abbott Laboratories, was identified through an affinity-based screening platform that sought to isolate JNK-targeting candidates from a library of 500 000 small molecules [46]. Whilst NMR revealed that the majority of candidate small molecules interacted with the ATP-binding site, two biaryl tetrazole compounds (including 46A, pubchem ID 15658026) elicited distinct resonance patterns and were found to bind a unique surface pocket bordered by the A-loop; the structural component that encompasses the TPY motif for MAP2K dual phosphorylation and activation (Figure 2B-ii). Structural analyses revealed that this binding pocket is accessible when JNK is inactive, due to mutations within the TPY motif (Thr183Glu and Tyr183Glu), causing distinct conformational changes in the A-loop. Accordingly, these compounds and their cell-active derivatives block JNK activity at low micro-molar ranges by inhibiting the phosphorylation of JNK by upstream MAP2Ks. To our knowledge, these compounds have not been investigated further in the context of JNK inhibition.

## Substrate-competitive JNK inhibitors

The best-described non-kinase strategy targets the JNK docking site (D-site), which lies next to the hinge loop and comprises the CD region and adjacent hydrophobic docking groove (Figure 2A). Much like the other MAPKs, P38 kinases and extracellular signal-regulated kinases (ERKs), JNKs use this single interface as a means of interacting with many of their binding partners, including scaffold proteins, upstream MAP2Ks, downstream substrates and phosphatases (reviewed in detail in [6]). These interaction partners harbor complimentary docking motifs (D-motifs or D-domains), which are typically found in disordered regions of the protein and contain basic residues (θ), followed by short spacer sequences and hydrophobic residues (φ). Two predominant types of JNK-interacting D-motifs have been identified [6], which resemble the sequences of the transcription factor N-FAT4 (θ-X-X-φ-X-φ-X-φ) or the JNK scaffold protein JNK-interacting protein 1 (JIP1) (θ-φ-X-X-φ-X-φ).

**Figure 3.**
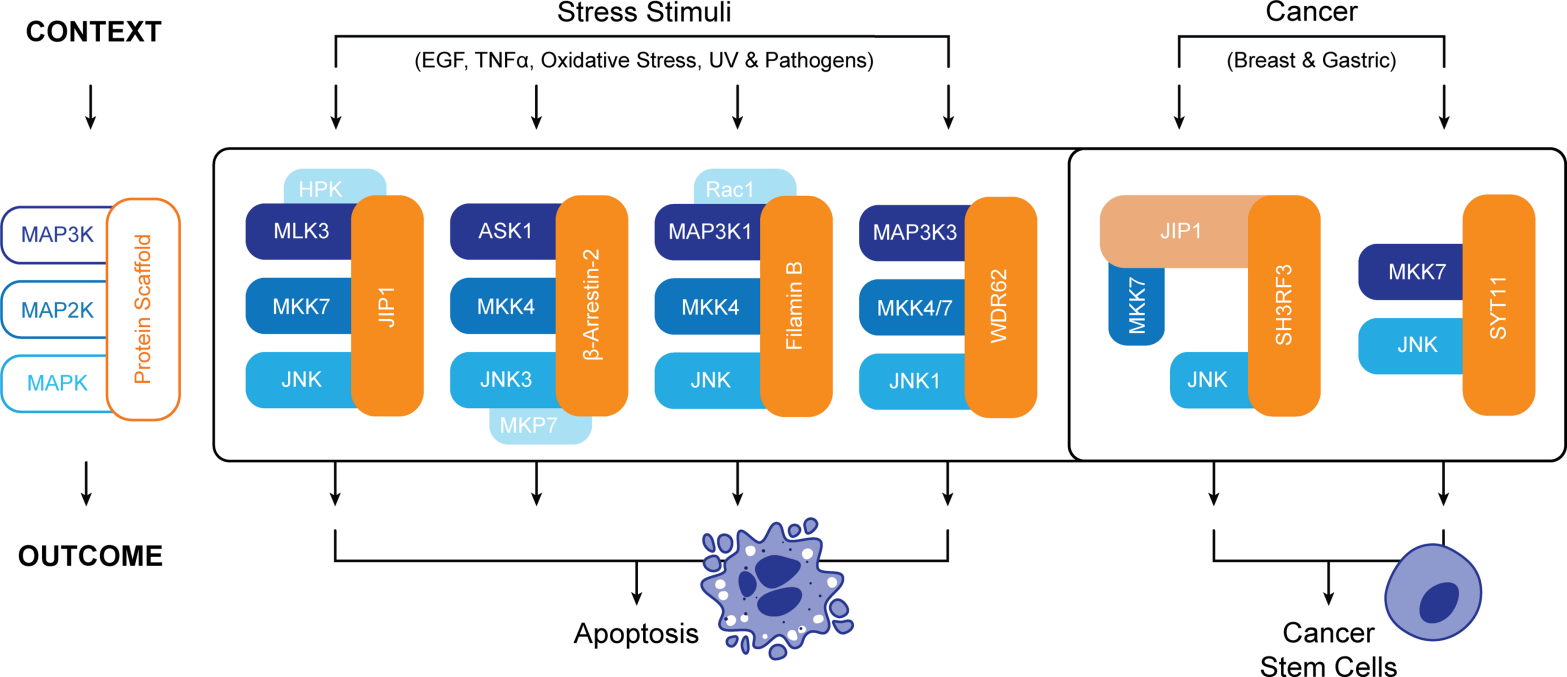
Scaffold proteins assemble compositionally and functionally discrete JNK signaling complexes in a highly context specific manner. JIP1, β-Arrestin-2, Filamin B and WDR62 are amongst the best described JNK scaffold proteins and are known to assemble JNK network components in response to extracellular stress stimuli to induce apoptosis. In the context of gastric and breast cancers, two scaffold proteins, Synaptotagmin 11 (SYT11) and SH3RF3, have recently been shown to promote disease progression through the positive regulation of cancer stem cell populations.

JIP1 is a prototypical scaffold protein in that it facilitates JNK signal transduction by binding all three components of the MAPK cascade, including MAP3Ks of the mixed lineage kinase (MLK) family, MKK7 and JNK (Figure 3) [47]. Significant interest in JIP1 stems from early reports that the overexpression of either JIP1 or the JIP1 JNK binding domain (JBD) is sufficient to block JNK activity [48,49]. These findings led to the development of the D-site targeting peptides pepJIP1 (also known as TI-JIP1; Figure 2B-iii), an 11 amino acid peptide corresponding to the minimal D-motif of JIP1 (residues 153–166) [50,51], and D-JNKI1, a cell-permeable and protease resistant retro-inverso peptide comprising the 20 amino acid JIP1 D-motif and a HIV-TAT sequence [52]. Whilst biochemical analyses reveal that pepJIP1 displays high specificity for JNKs, although with limited isoform selectivity, D-JNKI1 reportedly exhibits higher potency toward P38 kinases than JNKs [53,54]. In spite of this, the JNK inhibiting effects of D-JNKI1 have been assessed in various disease contexts including cancer, where it has been shown to suppress the growth of xenografted HCC cells and chemically induced liver cancers [55], and attenuate tumor growth and cancer pain development in murine melanoma models [56]. Although D-JNKI1 has not progressed as a clinical cancer treatment, its JNK-dependent otoprotective effects (reviewed in [57]) have prompted several preclinical studies and Phase II/III clinical trials evaluating its efficacy as a treatment for acute hearing loss [58,59]. It is likely that local intratympanic administration of D-JNKI1 (also known as AM-111 or brimapititde) in this clinical context may mitigate the toxic side effects of systemic JNK inhibition.

Whilst significant focus has been placed on JIP1-like peptides due to their high affinity interaction with JNK, peptide inhibitors have also been derived from the JNK docking protein and substrate, SH3 domain-binding protein 5 (SH3BP-5, or Sab). Expressed on the outer mitochondrial membrane, Sab interacts with and docks activated JNK through its cytoplasmic C-terminal kinase interaction domain (KIM), facilitating its translocation into mitochondria [60,61]. Mitochondrial JNK activity then drives Bcl-xl phosphorylation, cytochrome C release and respiration suppression, which ultimately activate processes such as apoptosis and autophagy [62–66]. Despite containing the essential D-motif components required for JNK docking, Sab has a significantly lower affinity for JNK compared with JIP1 [67,68]. Whilst it was originally thought that this lower affinity interaction would limit the use of Sab-derived peptides, such as TAT-Sab_KIM1_, it has instead been shown to increase their specificity for mitochondrial JNK [69]. Specifically, TAT-Sab_KIM1_ is able to inhibit JNK localization to mitochondria and mitochondrial JNK signaling pathways without perturbing cytosolic and nuclear JNK activation [66]. Whilst these peptides represent promising therapeutic targets in diseases where mitochondrial dysfunction is a key driver [64,66], the essential role of mitochondrial JNK in therapy-induced apoptotic response likely limits their use as cancer treatments. For instance, blocking the Sab-JNK interaction with TAT-Sab_KIM1_ has already been shown to prevent apoptosis induced by oxidative stress, including that driven by the kinase inhibitor sorafenib in HCC cells [70].

Several small molecule inhibitors of this JIP1-JNK interface have also been identified through large-scale compound screens. For instance, Stebbins et al. [71] utilized a dissociation enhanced lanthanide fluorescent assay to screen 30 000 compounds and identify BI-78D3, a highly specific and potent substrate-competitive JNK inhibitor that can sensitize osteosarcoma cells to doxorubicin treatment *in vitro* [72]. An alternate high-throughput screen of >2 million compounds performed by Pfizer identified an undisclosed number of highly specific JNK inhibitors in this same compound class, although their precise binding site could not be resolved [53]. This study additionally revealed a subset of dual-inhibitors that perturb both the JIP1–JNK and ATP-binding sites through allosteric mechanisms [53]. Based on the behavior of these dual-inhibitors in the presence of excess ATP, and their anticipated binding modes, it was predicted that they elicited their effects through interactions with the ATP-binding site rather than the D-site. Structural and biochemical analyses have since revealed the effects of both substrate-competitive JNK peptides and structurally diverse ATP-competitive compounds on this allosteric communication pathway between the JNK D-site, ATP-binding site and A-loop [68,73]. To our knowledge, no small molecule substrate-competitive JNK inhibitors or dual-inhibitors have progressed through to preclinical testing.

A significant limitation of strategies targeting the JNK D-site appears to be the fact that high-affinity inhibitors end up behaving much like ATP-competitive inhibitors, in that they effectively block all JNK-protein interactions and as such indiscriminately inhibit all JNK functions. Although studies assessing TAT-Sab_KIM1_ peptides on mitochondrial JNK indicate that specificity for discrete subcellular JNK pools can be achieved with low affinity inhibitors, it remains to be seen how low-affinity D-site targeting peptides would translate into a clinical setting given that they rely on subtle differences in interaction partner binding affinities to effectively target one JNK pool over another. Regardless, these peptides provide a crucial proof-of-principle that specific JNK functions can be perturbed by targeting protein-protein interaction interfaces within discrete JNK signaling complexes.

## Identifying alternate drug targets within JNK signaling complexes

Given the challenges faced with directly targeting JNK, a number of strategies have been described that perturb JNK signaling through alternate mechanisms. Amongst these is the recent development of specific MAP2K and MAP3K inhibitors, such as those targeting MKK7 [74], ASK1 [75], and TAK1 [76]. However, as each of these kinases is likely to regulate multiple downstream effectors and biological responses, it remains to be seen how they fare in terms of cellular toxicity. For instance, whilst MKK7 has been shown to promote the stemness of gastric cancers [77] and drive the metastasis of colon cancer cells [78], it also functions as a critical tumor suppressor in lung and mammary cancers through the stabilization of p53 [79]. Much like JNKs, the roles of MAP2Ks and MAP3Ks in tumorigenesis are highly context specific and a better understanding of their involvement in JNK signaling is required for the development of effective treatment strategies.

Although the JIP1/Sab-derived peptides demonstrate that protein-protein interactions represent attractive and effective therapeutic targets, there has been little progress targeting alternate interfaces within JNK signaling complexes. In one case, a 22 amino acid peptide identified through a fragment library screen, known as PYC71, was shown to bind to c-Jun and potently inhibit its interaction with JNKs [80]. This idea of targeting the D-motif interface rather than the D-site itself can also be seen in nature, with Notch1 able to block UV-irradiation induced JNK3 activation through its direct interaction with the JNK binding domain of JIP1 [81]. Whilst these studies support the idea of pursuing alternate protein targets, the identification of complex components (scaffolds and substrates) and interaction interfaces implicated in specific pro-tumorigenic JNK signaling pathways is not a trivial task.

Through their interactions with at least two components of the MAPK tier, scaffold proteins fine-tune the composition and localization of JNK signaling complexes to enhance the specificity and efficacy of signal transduction. Whilst a number of JNK scaffolds have been identified, including JIP family members [82], arrestins [83,84], filamins [85,86] and WDR62 [87,88] (Figure 3), their JNK-related roles have almost exclusively been linked to stress-induced apoptosis and limited literature is available covering their relevance as scaffolds in cancer progression. To this end, SH3 domain containing ring finger 3 (SH3RF3, also POSH2) and Synaptotagmin 11 (SYT11) have both recently been identified as JNK scaffolds that positively regulate cancer stem cells (CSCs) in breast and gastric cancers [14,77]. CSCs are a tumor cell subpopulation that are capable of self-renewal, display tumor-initiating capabilities and are associated with metastasis, therapy resistance and disease recurrence. Recent studies have now demonstrated that JNK signaling is critical for driving the transcriptional programs that maintain CSC phenotypes in multiple cancer contexts [89–93], and also shown that JNK promotes CSC chemoresistance and metastasis in TNBCs by supporting the formation of a CSC niche [12].

With protein-protein interactions partly described for both the SH3RF3 and SYT11 JNK signaling complexes, it is clear that whilst these two scaffolds drive similar biological outcomes, they each regulate JNK through distinct mechanisms [14,77] (Figure 3). In the context of gastric cancer, Kim *et al.* demonstrated that both MKK7 and JNK interact with the SYT11 in the cytoplasm via its N-terminal transmembrane domain. SYT11-dependent JNK phosphorylation in turn activates c-Jun and the subsequent transcription of EMT-related genes that drive tumor formation and liver metastasis [77]. Alternately, Zhang et al. reported that whist MKK7 directly binds the fourth SH3 domain of SH3RF3, JIP1 is required to mediate the interaction between SH3RF3 and JNK. JNK phosphorylation in this context promotes breast CSC phenotypes through the downstream activation of c-Jun and increased expression of pentraxin 3 (PTX3) [14]. In both cases, JNK inhibition achieved through scaffold depletion or small-molecule inhibitors significantly perturbed oncogenesis. Whilst further work is required to fully resolve these binding interfaces, the protein-protein interactions uncovered in these studies open up novel therapeutic options for targeting the discrete oncogenic JNK signaling complexes that regulate cancer stem cell populations in specific tumor types.

## Concluding remarks

Whilst JNK proteins play critical tumor-promoting roles in a number of cancers, their tumor-suppressive, homeostatic and apoptotic functions limit the use of direct JNK inhibitors as anti-cancer treatments. Despite the significant efforts that have been made concerning the development and optimization of ATP-competitive and substrate-competitive JNK inhibitors, it is increasingly clear that strategies targeting JNK directly are unlikely to yield clinical success as they indiscriminately block all JNK functions and lack the subtlety required to selectively suppress oncogenic JNK signaling. For the future of JNK targeting in cancer, we believe that this specificity can be achieved by inhibiting unique protein-protein interactions within oncogenic JNK signaling complexes, including the interactions between scaffold proteins and MAP3K/MAP2K/JNK network components. Whilst recent studies have made progress in identifying oncogenic JNK complexes that regulate cancer stem cell populations in gastric and breast cancers, significantly more work is required to therapeutically target these complexes, and resolve the composition, organization and interfaces of JNK complexes that drive tumorigenesis and metastatic disease progression in other tumor types.

## Perspectives

- JNKs represent attractive therapeutic targets for several cancers, including gastric and breast cancers. Various experimental models have demonstrated that direct JNK inhibition is able to block primary tumor growth, impede the tumor-initiating potential of cancer stem cells, modulate the structural and cellular landscape of the metastatic niche, and thereby inhibit metastatic disease progression.
- Although significant headway has been made in improving the specificity and selectively of ATP-competitive and substrate-competitive JNK inhibitors, these compounds and peptides are unlikely to yield clinical success as anti-cancer therapies due to their indiscriminate inhibitory effects on the physiological and tumor-suppressing functions of JNK. The evidence indicates that direct JNK inhibition is unlikely to work and that alternate approaches are required. In line with this, strategies targeting the scaffold-JNK and JNK-substrate interfaces provide a crucial proof-of-principle that protein–protein interactions within discrete JNK signaling complexes represent effective and clinically viable therapeutic targets.
- For clinical viability, anti-cancer therapies targeting JNK must discriminate between the distinct functions of JNK and specifically inhibit JNKs oncogenic activities. Identifying the scaffold proteins, substrates and protein–protein interaction interfaces that drive oncogenic JNK signaling will be critical for the development of effective JNK-targeting strategies in these disease contexts.

## Abbreviations

CSC: cancer stem cell
D-motif: docking motif (JNK interaction partner)
D-site: docking site (JNK)
ERK: extracellular signal-regulated kinases
HCC: hepatocellular carcinoma
JBD: JNK binding doamin
JIP: JNK-interacting protein
JNK: c-Jun N-terminal kinase
MAP2K: mitogen activated protein kinase kinase
MAP3K: mitogen activated protein kinase kinase kinase
MAPK: mitogen activated protein kinase
SAB/SH3BP-5: SH3 domain-binding protein 5
SAPK: stress activated protein kinase
SH3RF3: SH3 domain containing ring finger 3
SYT11: Synaptotagmin 11
TNBC: triple negative breast cancer

## References

[1] Davis, R.J. (2000) Signal transduction by the JNK group of MAP kinases. Cell 103, 239–252 10.1016/s0092-8674(00)00116-1 11057897

[2] Wagner, E.F. and Nebreda, A.R. (2009) Signal integration by JNK and p38 MAPK pathways in cancer development. Nat. Rev. Cancer 9, 537–549 10.1038/nrc2694 19629069

[3] Bubici, C. and Papa, S. (2014) JNK signalling in cancer: in need of new, smarter therapeutic targets. Br. J. Pharmacol. 171, 24–37 10.1111/bph.12432 24117156 PMC3874694

[4] Engstrom, W., Ward, A. and Moorwood, K. (2010) The role of scaffold proteins in JNK signalling. Cell Prolif. 43, 56–66 10.1111/j.1365-2184.2009.00654.x 19922489 PMC6496263

[5] Witzel, F., Maddison, L. and Bluthgen, N. (2012) How scaffolds shape MAPK signaling: what we know and opportunities for systems approaches. Front. Physiol. 3, 475 10.3389/fphys.2012.00475 23267331 PMC3527831

[6] Zeke, A., Misheva, M., Remenyi, A. and Bogoyevitch, M.A. (2016) JNK signaling: regulation and functions based on complex protein-protein partnerships. Microbiol. Mol. Biol. Rev. 80, 793–835 10.1128/MMBR.00043-14 27466283 PMC4981676

[7] Tournier, C. (2013) The 2 faces of JNK signaling in cancer. Genes Cancer 4, 397–400 10.1177/1947601913486349 24349637 PMC3863340

[8] Cellurale, C., Girnius, N., Jiang, F., Cavanagh-Kyros, J., Lu, S., Garlick, D.S. et al. (2012) Role of JNK in mammary gland development and breast cancer. Cancer Res. 72, 472–481 10.1158/0008-5472.CAN-11-1628 22127926 PMC3261359

[9] Girnius, N., Edwards, Y.J.K. and Davis, R.J. (2018) The cJUN NH2-terminal kinase (JNK) pathway contributes to mouse mammary gland remodeling during involution. Cell Death Differ. 25, 1702–1715 10.1038/s41418-018-0081-z 29511338 PMC6143629

[10] Cellurale, C., Weston, C.R., Reilly, J., Garlick, D.S., Jerry, D.J., Sluss, H.K. et al. (2010) Role of JNK in a Trp53-dependent mouse model of breast cancer. PLoS ONE 5, e12469 10.1371/journal.pone.0012469 20814571 PMC2930003

[11] Girnius, N., Edwards, Y.J., Garlick, D.S. and Davis, R.J. (2018) The cJUN NH2-terminal kinase (JNK) signaling pathway promotes genome stability and prevents tumor initiation. eLife 7, e36389 10.7554/eLife.36389 29856313 PMC5984035

[12] Insua-Rodriguez, J., Pein, M., Hongu, T., Meier, J., Descot, A., Lowy, C.M. et al. (2018) Stress signaling in breast cancer cells induces matrix components that promote chemoresistant metastasis. EMBO Mol. Med. 10, e9003 10.15252/emmm.201809003 30190333 PMC6180299

[13] Pein, M., Insua-Rodriguez, J., Hongu, T., Riedel, A., Meier, J., Wiedmann, L. et al. (2020) Metastasis-initiating cells induce and exploit a fibroblast niche to fuel malignant colonization of the lungs. Nat. Commun. 11, 1494 10.1038/s41467-020-15188-x 32198421 PMC7083860

[14] Zhang, P., Liu, Y., Lian, C., Cao, X., Wang, Y., Li, X. et al. (2020) SH3RF3 promotes breast cancer stem-like properties via JNK activation and PTX3 upregulation. Nat. Commun. 11, 2487 10.1038/s41467-020-16051-9 32427938 PMC7237486

[15] Semba, T., Wang, X., Xie, X., Cohen, E.N., Reuben, J.M., Dalby, K.N. et al. (2022) Identification of the JNK-active triple-Negative breast cancer cluster associated with an immunosuppressive tumor microenvironment. J. Natl Cancer Inst. 114, 97–108 10.1093/jnci/djab128 34250544 PMC8755499

[16] Ashenden, M., van Weverwijk, A., Murugaesu, N., Fearns, A., Campbell, J., Gao, Q. et al. (2017) An *in vivo* functional screen identifies JNK signaling as a modulator of chemotherapeutic response in breast cancer. Mol. Cancer Ther. 16, 1967–1978 10.1158/1535-7163.MCT-16-0731 28611109

[17] Messoussi, A., Feneyrolles, C., Bros, A., Deroide, A., Dayde-Cazals, B., Cheve, G. et al. (2014) Recent progress in the design, study, and development of c-Jun N-terminal kinase inhibitors as anticancer agents. Chem. Biol. 21, 1433–1443 10.1016/j.chembiol.2014.09.007 25442375

[18] Duong, M.T.H., Lee, J.H. and Ahn, H.C. (2020) C-Jun N-terminal kinase inhibitors: Structural insight into kinase-inhibitor complexes. Comput. Struct. Biotechnol. J. 18, 1440–1457 10.1016/j.csbj.2020.06.013 32637042 PMC7327381

[19] Bennett, B.L., Sasaki, D.T., Murray, B.W., O'Leary, E.C., Sakata, S.T., Xu, W. et al. (2001) SP600125, an anthrapyrazolone inhibitor of Jun N-terminal kinase. Proc. Natl Acad. Sci. U.S.A. 98, 13681–13686 10.1073/pnas.251194298 11717429 PMC61101

[20] Mingo-Sion, A.M., Marietta, P.M., Koller, E., Wolf, D.M. and Van Den Berg, C.L. (2004) Inhibition of JNK reduces G2/M transit independent of p53, leading to endoreduplication, decreased proliferation, and apoptosis in breast cancer cells. Oncogene 23, 596–604 10.1038/sj.onc.1207147 14724588

[21] Jacobs-Helber, S.M. and Sawyer, S.T. (2004) Jun N-terminal kinase promotes proliferation of immature erythroid cells and erythropoietin-dependent cell lines. Blood 104, 696–703 10.1182/blood-2003-05-1754 15059850

[22] Grassi, E.S., Vezzoli, V., Negri, I., Labadi, A., Fugazzola, L., Vitale, G. et al. (2015) SP600125 has a remarkable anticancer potential against undifferentiated thyroid cancer through selective action on ROCK and p53 pathways. Oncotarget 6, 36383–36399 10.18632/oncotarget.5799 26415230 PMC4742184

[23] Bain, J., McLauchlan, H., Elliott, M. and Cohen, P. (2003) The specificities of protein kinase inhibitors: an update. Biochem. J. 371, 199–204 10.1042/BJ20021535 12534346 PMC1223271

[24] Vasilevskaya, I.A., Selvakumaran, M., Hierro, L.C., Goldstein, S.R., Winkler, J.D. and O'Dwyer, P.J. (2015) Inhibition of JNK sensitizes hypoxic colon cancer cells to DNA-damaging agents. Clin. Cancer Res. 21, 4143–4152 10.1158/1078-0432.CCR-15-0352 26023085 PMC4573818

[25] Okada, M., Kuramoto, K., Takeda, H., Watarai, H., Sakaki, H., Seino, S. et al. (2016) The novel JNK inhibitor AS602801 inhibits cancer stem cells in vitro and in vivo. Oncotarget 7, 27021–27032 10.18632/oncotarget.8395 27027242 PMC5053629

[26] Kuramoto, K., Yamamoto, M., Suzuki, S., Sanomachi, T., Togashi, K., Seino, S. et al. (2018) AS602801, an anti-cancer stem cell drug candidate, suppresses gap-junction communication between lung cancer stem cells and astrocytes. Anticancer Res. 38, 5093–5099 10.21873/anticanres.12829 30194154

[27] Yamamoto, M., Suzuki, S., Togashi, K., Sanomachi, T., Seino, S., Kitanaka, C. et al. (2019) AS602801 sensitizes ovarian cancer stem cells to paclitaxel by down-regulating MDR1. Anticancer Res. 39, 609–617 10.21873/anticanres.13154 30711936

[28] Li, Z., Sun, C., Tao, S., Osunkoya, A.O., Arnold, R.S., Petros, J.A. et al. (2020) The JNK inhibitor AS602801 synergizes with enzalutamide to kill prostate cancer cells *in vitro* and *in vivo* and inhibit androgen receptor expression. Transl. Oncol. 13, 100751 10.1016/j.tranon.2020.100751 32199273 PMC7082632

[29] Zhang, S., Gong, Y., Wang, H., Li, Z., Huang, Y., Fu, X. et al. (2021) AS602801 sensitizes glioma cells to temozolomide and vincristine by blocking gap junction communication between glioma cells and astrocytes. J. Cell. Mol. Med. 25, 4062–4072 10.1111/jcmm.16375 33609076 PMC8051707

[30] Zhang, T., Inesta-Vaquera, F., Niepel, M., Zhang, J., Ficarro, S.B., Machleidt, T. et al. (2012) Discovery of potent and selective covalent inhibitors of JNK. Chem. Biol. 19, 140–154 10.1016/j.chembiol.2011.11.010 22284361 PMC3270411

[31] Ebelt, N.D., Kaoud, T.S., Edupuganti, R., Van Ravenstein, S., Dalby, K.N. and Van Den Berg, C.L. (2017) A c-Jun N-terminal kinase inhibitor, JNK-IN-8, sensitizes triple negative breast cancer cells to lapatinib. Oncotarget 8, 104894–104912 10.18632/oncotarget.20581 29285221 PMC5739608

[32] Lipner, M.B., Peng, X.L., Jin, C., Xu, Y., Gao, Y., East, M.P. et al. (2020) Irreversible JNK1-JUN inhibition by JNK-IN-8 sensitizes pancreatic cancer to 5-FU/FOLFOX chemotherapy. JCI Insight 5, e129905 10.1172/jci.insight.129905 32213714 PMC7205424

[33] Hubner, A., Mulholland, D.J., Standen, C.L., Karasarides, M., Cavanagh-Kyros, J., Barrett, T. et al. (2012) JNK and PTEN cooperatively control the development of invasive adenocarcinoma of the prostate. Proc. Natl Acad. Sci. U.S.A. 109, 12046–12051 10.1073/pnas.1209660109 22753496 PMC3409732

[34] Ebelt, N.D. and Cantrell, M.A. (2013) Van Den Berg CL. c-Jun N-Terminal kinases mediate a wide range of targets in the metastatic cascade. Genes Cancer 4, 378–387 10.1177/1947601913485413 24349635 PMC3863335

[35] Christopher, J.A., Atkinson, F.L., Bax, B.D., Brown, M.J., Champigny, A.C., Chuang, T.T. et al. (2009) 1-Aryl-3,4-dihydroisoquinoline inhibitors of JNK3. Bioorg. Med. Chem. Lett. 19, 2230–2234 10.1016/j.bmcl.2009.02.098 19303774

[36] Kamenecka, T., Habel, J., Duckett, D., Chen, W., Ling, Y.Y., Frackowiak, B. et al. (2009) Structure-activity relationships and X-ray structures describing the selectivity of aminopyrazole inhibitors for c-Jun N-terminal kinase 3 (JNK3) over p38. J. Biol. Chem. 284, 12853–12861 10.1074/jbc.M809430200 19261605 PMC2676016

[37] Zheng, K., Iqbal, S., Hernandez, P., Park, H., LoGrasso, P.V. and Feng, Y. (2014) Design and synthesis of highly potent and isoform selective JNK3 inhibitors: SAR studies on aminopyrazole derivatives. J. Med. Chem. 57, 10013–10030 10.1021/jm501256y 25393557 PMC4266361

[38] Plantevin Krenitsky, V., Nadolny, L., Delgado, M., Ayala, L., Clareen, S.S., Hilgraf, R. et al. (2012) Discovery of CC-930, an orally active anti-fibrotic JNK inhibitor. Bioorg. Med. Chem. Lett. 22, 1433–1438 10.1016/j.bmcl.2011.12.027 22244937

[39] Nagy, M.A., Hilgraf, R., Mortensen, D.S., Elsner, J., Norris, S., Tikhe, J. et al. (2021) Discovery of the c-Jun N-terminal kinase inhibitor CC-90001. J. Med. Chem. 64, 18193–18208 10.1021/acs.jmedchem.1c01716 34894681

[40] van der Velden, J.L., Ye, Y., Nolin, J.D., Hoffman, S.M., Chapman, D.G., Lahue, K.G. et al. (2016) JNK inhibition reduces lung remodeling and pulmonary fibrotic systemic markers. Clin. Transl. Med. 5, 36 10.1186/s40169-016-0117-2 27590145 PMC5010551

[41] Tong, Z., Gaudy, A., Tatosian, D., Ramirez-Valle, F., Liu, H., Chen, J. et al. (2021) Assessment of drug-drug interactions of CC-90001, a potent and selective inhibitor of c-Jun N-terminal kinase. Xenobiotica 51, 1416–1426 10.1080/00498254.2022.2027553 35000550

[42] Popmihajlov, Z., Sutherland, D.J., Horan, G.S., Ghosh, A., Lynch, D.A., Noble, P.W. et al. (2022) CC-90001, a c-Jun N-terminal kinase (JNK) inhibitor, in patients with pulmonary fibrosis: design of a phase 2, randomised, placebo-controlled trial. BMJ Open Respir. Res. 9, e001060 10.1136/bmjresp-2021-001060 PMC8783810 35058236

[43] Chang, Q., Zhang, Y., Beezhold, K.J., Bhatia, D., Zhao, H., Chen, J. et al. (2009) Sustained JNK1 activation is associated with altered histone H3 methylations in human liver cancer. J. Hepatol. 50, 323–333 10.1016/j.jhep.2008.07.037 19041150 PMC4417500

[44] Chang, Q., Chen, J., Beezhold, K.J., Castranova, V., Shi, X. and Chen, F. (2009) JNK1 activation predicts the prognostic outcome of the human hepatocellular carcinoma. Mol. Cancer 8, 64 10.1186/1476-4598-8-64 19686584 PMC2732591

[45] Takahashi, H., Ogata, H., Nishigaki, R., Broide, D.H. and Karin, M. (2010) Tobacco smoke promotes lung tumorigenesis by triggering IKKbeta- and JNK1-dependent inflammation. Cancer Cell 17, 89–97 10.1016/j.ccr.2009.12.008 20129250 PMC2818776

[46] Comess, K.M., Sun, C., Abad-Zapatero, C., Goedken, E.R., Gum, R.J., Borhani, D.W. et al. (2011) Discovery and characterization of non-ATP site inhibitors of the mitogen activated protein (MAP) kinases. ACS Chem. Biol. 6, 234–244 10.1021/cb1002619 21090814

[47] Whitmarsh, A.J., Cavanagh, J., Tournier, C., Yasuda, J. and Davis, R.J. (1998) A mammalian scaffold complex that selectively mediates MAP kinase activation. Science 281, 1671–1674 10.1126/science.281.5383.1671 9733513

[48] Dickens, M., Rogers, J.S., Cavanagh, J., Raitano, A., Xia, Z., Halpern, J.R. et al. (1997) A cytoplasmic inhibitor of the JNK signal transduction pathway. Science 277, 693–696 10.1126/science.277.5326.693 9235893

[49] Ammendrup, A., Maillard, A., Nielsen, K., Aabenhus Andersen, N., Serup, P., Dragsbaek Madsen, O. et al. (2000) The c-Jun amino-terminal kinase pathway is preferentially activated by interleukin-1 and controls apoptosis in differentiating pancreatic beta-cells. Diabetes 49, 1468–1476 10.2337/diabetes.49.9.1468 10969830

[50] Barr, R.K., Kendrick, T.S. and Bogoyevitch, M.A. (2002) Identification of the critical features of a small peptide inhibitor of JNK activity. J. Biol. Chem. 277, 10987–10997 10.1074/jbc.M107565200 11790767

[51] Heo, Y.S., Kim, S.K., Seo, C.I., Kim, Y.K., Sung, B.J., Lee, H.S. et al. (2004) Structural basis for the selective inhibition of JNK1 by the scaffolding protein JIP1 and SP600125. EMBO J. 23, 2185–2195 10.1038/sj.emboj.7600212 15141161 PMC419904

[52] Bonny, C., Oberson, A., Negri, S., Sauser, C. and Schorderet, D.F. (2001) Cell-permeable peptide inhibitors of JNK: novel blockers of beta-cell death. Diabetes 50, 77–82 10.2337/diabetes.50.1.77 11147798

[53] Chen, T., Kablaoui, N., Little, J., Timofeevski, S., Tschantz, W.R., Chen, P. et al. (2009) Identification of small-molecule inhibitors of the JIP-JNK interaction. Biochem. J. 420, 283–294 10.1042/BJ20081899 19243309

[54] Kaoud, T.S., Mitra, S., Lee, S., Taliaferro, J., Cantrell, M., Linse, K.D. et al. (2011) Development of JNK2-selective peptide inhibitors that inhibit breast cancer cell migration. ACS Chem. Biol. 6, 658–666 10.1021/cb200017n 21438496 PMC3401522

[55] Hui, L., Zatloukal, K., Scheuch, H., Stepniak, E. and Wagner, E.F. (2008) Proliferation of human HCC cells and chemically induced mouse liver cancers requires JNK1-dependent p21 downregulation. J. Clin. Invest. 118, 3943–3953 10.1172/JCI37156 19033664 PMC2579707

[56] Gao, Y.J., Cheng, J.K., Zeng, Q., Xu, Z.Z., Decosterd, I., Xu, X. et al. (2009) Selective inhibition of JNK with a peptide inhibitor attenuates pain hypersensitivity and tumor growth in a mouse skin cancer pain model. Exp. Neurol. 219, 146–155 10.1016/j.expneurol.2009.05.006 19445931 PMC2728781

[57] Eshraghi, A.A., Aranke, M., Salvi, R., Ding, D., Coleman, Jr, J.K.M., Ocak, E. et al. (2018) Preclinical and clinical otoprotective applications of cell-penetrating peptide D-JNKI-1 (AM-111). Hear. Res. 368, 86–91 10.1016/j.heares.2018.03.003 29573879

[58] Suckfuell, M., Lisowska, G., Domka, W., Kabacinska, A., Morawski, K., Bodlaj, R. et al. (2014) Efficacy and safety of AM-111 in the treatment of acute sensorineural hearing loss: a double-blind, randomized, placebo-controlled phase II study. Otol. Neurotol. 35, 1317–1326 10.1097/MAO.0000000000000466 24979398

[59] Staecker, H., Jokovic, G., Karpishchenko, S., Kienle-Gogolok, A., Krzyzaniak, A., Lin, C.D. et al. (2019) Efficacy and safety of AM-111 in the treatment of acute unilateral sudden deafness-a double-blind, randomized, placebo-controlled phase 3 study. Otol. Neurotol. 40, 584–594 10.1097/MAO.0000000000002229 31083077 PMC6553962

[60] Wiltshire, C., Matsushita, M., Tsukada, S., Gillespie, D.A. and May, G.H. (2002) A new c-Jun N-terminal kinase (JNK)-interacting protein, Sab (SH3BP5), associates with mitochondria. Biochem. J. 367, 577–585 10.1042/BJ20020553 12167088 PMC1222945

[61] Win, S., Than, T.A. and Kaplowitz, N. (2018) The regulation of JNK signaling pathways in cell death through the interplay with mitochondrial SAB and upstream post-Translational effects. Int. J. Mol. Sci. 19, 3657 10.3390/ijms19113657 30463289 PMC6274687

[62] Chambers, J.W. and LoGrasso, P.V. (2011) Mitochondrial c-Jun N-terminal kinase (JNK) signaling initiates physiological changes resulting in amplification of reactive oxygen species generation. J. Biol. Chem. 286, 16052–16062 10.1074/jbc.M111.223602 21454558 PMC3091214

[63] Win, S., Than, T.A., Fernandez-Checa, J.C. and Kaplowitz, N. (2014) JNK interaction with Sab mediates ER stress induced inhibition of mitochondrial respiration and cell death. Cell Death Dis. 5, e989 10.1038/cddis.2013.522 24407242 PMC4040675

[64] Xu, J., Qin, X., Cai, X., Yang, L., Xing, Y., Li, J. et al. (2015) Mitochondrial JNK activation triggers autophagy and apoptosis and aggravates myocardial injury following ischemia/reperfusion. Biochim. Biophys. Acta 1852, 262–270 10.1016/j.bbadis.2014.05.012 24859228

[65] Win, S., Than, T.A., Min, R.W. and Aghajan, M. (2016) Kaplowitz N. c-Jun N-terminal kinase mediates mouse liver injury through a novel Sab (SH3BP5)-dependent pathway leading to inactivation of intramitochondrial Src. Hepatology 63, 1987–2003 10.1002/hep.28486 26845758 PMC4874901

[66] Li, C., Ma, D., Chen, Y., Liu, W., Jin, F. and Bo, L. (2022) Selective inhibition of JNK located on mitochondria protects against mitochondrial dysfunction and cell death caused by endoplasmic reticulum stress in mice with LPSinduced ALI/ARDS. Int. J. Mol. Med. 49, 85 10.3892/ijmm.2022.5141 35514298 PMC9106374

[67] Barr, R.K., Boehm, I., Attwood, P.V., Watt, P.M. and Bogoyevitch, M.A. (2004) The critical features and the mechanism of inhibition of a kinase interaction motif-based peptide inhibitor of JNK. J. Biol. Chem. 279, 36327–36338 10.1074/jbc.M402181200 15208323

[68] Laughlin, J.D., Nwachukwu, J.C., Figuera-Losada, M., Cherry, L., Nettles, K.W. and LoGrasso, P.V. (2012) Structural mechanisms of allostery and autoinhibition in JNK family kinases. Structure 20, 2174–2184 10.1016/j.str.2012.09.021 23142346 PMC3589125

[69] Chambers, J.W., Cherry, L., Laughlin, J.D., Figuera-Losada, M. and Lograsso, P.V. (2011) Selective inhibition of mitochondrial JNK signaling achieved using peptide mimicry of the Sab kinase interacting motif-1 (KIM1). ACS Chem. Biol. 6, 808–818 10.1021/cb200062a 21563797 PMC3158843

[70] Heslop, K.A., Rovini, A., Hunt, E.G. Fang, D., Morris, M.E., Christie, C.F. et al. (2020) JNK activation and translocation to mitochondria mediates mitochondrial dysfunction and cell death induced by VDAC opening and sorafenib in hepatocarcinoma cells. Biochem. Pharmacol. 171, 113728 10.1016/j.bcp.2019.113728 31759978 PMC7309270

[71] Stebbins, J.L., De, S.K., Machleidt, T., Becattini, B., Vazquez, J., Kuntzen, C. et al. (2008) Identification of a new JNK inhibitor targeting the JNK-JIP interaction site. Proc. Natl Acad. Sci. U.S.A. 105, 16809–16813 10.1073/pnas.0805677105 18922779 PMC2567907

[72] Posthumadeboer, J., van Egmond, P.W., Helder, M.N., de Menezes, R.X., Cleton-Jansen, A.M., Belien, J.A. et al. (2012) Targeting JNK-interacting-protein-1 (JIP1) sensitises osteosarcoma to doxorubicin. Oncotarget 3, 1169–1181 10.18632/oncotarget.600 23045411 PMC3717953

[73] Lombard, C.K., Davis, A.L., Inukai, T. and Maly, D.J. (2018) Allosteric modulation of JNK docking site interactions with ATP-competitive inhibitors. Biochemistry 57, 5897–5909 10.1021/acs.biochem.8b00776 30211540 PMC6338552

[74] Schroder, M., Tan, L., Wang, J., Liang, Y., Gray, N.S., Knapp, S. et al. (2020) Catalytic domain plasticity of MKK7 reveals structural mechanisms of allosteric activation and diverse targeting opportunities. Cell Chem. Biol. 27, 1285–95.e4 10.1016/j.chembiol.2020.07.014 32783966

[75] Ogier, J.M., Nayagam, B.A. and Lockhart, P.J. (2020) ASK1 inhibition: a therapeutic strategy with multi-system benefits. J. Mol. Med. (Berl) 98, 335–348 10.1007/s00109-020-01878-y 32060587 PMC7080683

[76] Totzke, J., Gurbani, D., Raphemot, R., Hughes, P.F., Bodoor, K., Carlson, D.A. et al. (2017) Takinib, a selective TAK1 inhibitor, broadens the therapeutic efficacy of TNF-alpha inhibition for cancer and autoimmune disease. Cell Chem. Biol. 24, 1029–39 e7 10.1016/j.chembiol.2017.07.011 28820959 PMC5576570

[77] Kim, B.K., Kim, D.M., Park, H., Kim, S.K., Hwang, M.A., Lee, J. et al. (2022) Synaptotagmin 11 scaffolds MKK7-JNK signaling process to promote stem-like molecular subtype gastric cancer oncogenesis. J. Exp. Clin. Cancer Res. 41, 212 10.1186/s13046-022-02420-3 35768842 PMC9241269

[78] Sakai, H., Sato, A., Aihara, Y., Ikarashi, Y., Midorikawa, Y., Kracht, M. et al. (2014) MKK7 mediates miR-493-dependent suppression of liver metastasis of colon cancer cells. Cancer Sci. 105, 425–430 10.1111/cas.12380 24533778 PMC4317799

[79] Schramek, D., Kotsinas, A., Meixner, A., Wada, T., Elling, U., Pospisilik, J.A. et al. (2011) The stress kinase MKK7 couples oncogenic stress to p53 stability and tumor suppression. Nat. Genet. 43, 212–219 10.1038/ng.767 21317887

[80] Ngoei, K.R., Catimel, B., Church, N., Lio, D.S., Dogovski, C., Perugini, M.A. et al. (2011) Characterization of a novel JNK (c-Jun N-terminal kinase) inhibitory peptide. Biochem. J. 434, 399–413 10.1042/BJ20101244 21162712

[81] Kim, J.W., Kim, M.J., Kim, K.J., Yun, H.J., Chae, J.S., Hwang, S.G. et al. (2005) Notch interferes with the scaffold function of JNK-interacting protein 1 to inhibit the JNK signaling pathway. Proc. Natl Acad. Sci. U.S.A. 102, 14308–14313 10.1073/pnas.0501600102 16179393 PMC1242280

[82] Whitmarsh, A.J. (2006) The JIP family of MAPK scaffold proteins. Biochem. Soc. Trans. 34, 828–832 10.1042/BST0340828 17052208

[83] McDonald, P.H., Chow, C.W., Miller, W.E., Laporte, S.A., Field, M.E., Lin, F.T. et al. (2000) Beta-arrestin 2: a receptor-regulated MAPK scaffold for the activation of JNK3. Science 290, 1574–1577 10.1126/science.290.5496.1574 11090355

[84] Zhan, X., Kook, S., Gurevich, E.V. and Gurevich, V.V. (2014) Arrestin-dependent activation of JNK family kinases. Handb. Exp. Pharmacol. 219, 259–280 10.1007/978-3-642-41199-1_13 24292834 PMC4514028

[85] Marti, A., Luo, Z., Cunningham, C., Ohta, Y., Hartwig, J., Stossel, T.P. et al. (1997) Actin-binding protein-280 binds the stress-activated protein kinase (SAPK) activator SEK-1 and is required for tumor necrosis factor-alpha activation of SAPK in melanoma cells. J. Biol. Chem. 272, 2620–2628 10.1074/jbc.272.5.2620 9006895

[86] Jeon, Y.J., Choi, J.S., Lee, J.Y., Yu, K.R., Ka, S.H., Cho, Y. et al. (2008) Filamin B serves as a molecular scaffold for type I interferon-induced c-Jun NH2-terminal kinase signaling pathway. Mol. Biol. Cell 19, 5116–5130 10.1091/mbc.E08-06-0576 18815275 PMC2592671

[87] Wasserman, T., Katsenelson, K., Daniliuc, S., Hasin, T., Choder, M. and Aronheim, A. (2010) A novel c-Jun N-terminal kinase (JNK)-binding protein WDR62 is recruited to stress granules and mediates a nonclassical JNK activation. Mol. Biol. Cell 21, 117–130 10.1091/mbc.E09-06-0512 19910486 PMC2801705

[88] Prinz, E., Aviram, S. and Aronheim, A. (2018) WDR62 mediates TNFalpha-dependent JNK activation via TRAF2-MLK3 axis. Mol. Biol. Cell 29, 2470–2480 10.1091/mbc.E17-08-0504 30091641 PMC6233063

[89] Yoon, C.H., Kim, M.J., Kim, R.K., Lim, E.J., Choi, K.S., An, S. et al. (2012) . c-Jun N-terminal kinase has a pivotal role in the maintenance of self-renewal and tumorigenicity in glioma stem-like cells. Oncogene 31, 4655–4666 10.1038/onc.2011.634 22249269

[90] Tong, M., Fung, T.M., Luk, S.T., Ng, K.Y., Lee, T.K., Lin, C.H. et al. (2015) ANXA3/JNK signaling promotes self-renewal and tumor growth, and its blockade provides a therapeutic target for hepatocellular carcinoma. Stem Cell Rep. 5, 45–59 10.1016/j.stemcr.2015.05.013 PMC4618447 26095609

[91] Xie, X., Kaoud, T.S., Edupuganti, R., Zhang, T., Kogawa, T., Zhao, Y. et al. (2017) . c-Jun N-terminal kinase promotes stem cell phenotype in triple-negative breast cancer through upregulation of Notch1 via activation of c-Jun. Oncogene 36, 2599–2608 10.1038/onc.2016.417 27941886 PMC6116358

[92] Fang, M., Li, Y., Huang, K., Qi, S., Zhang, J., Zgodzinski, W. et al. (2017) IL33 promotes colon cancer cell stemness via JNK activation and macrophage recruitment. Cancer Res. 77, 2735–2745 10.1158/0008-5472.CAN-16-1602 28249897 PMC5760170

[93] Hao, P., Zhang, J., Fang, S., Jia, M., Xian, X., Yan, S. et al. (2022) Lipocalin-2 inhibits pancreatic cancer stemness via the AKT/c-Jun pathway. Hum. Cell 35, 1475–1486 10.1007/s13577-022-00735-z 35792978

[94] Jumper, J., Evans, R., Pritzel, A., Green, T., Figurnov, M., Ronneberger, O. et al. (2021) Highly accurate protein structure prediction with AlphaFold. Nature 596, 583–589 10.1038/s41586-021-03819-2 34265844 PMC8371605

[95] Varadi, M., Anyango, S., Deshpande, M., Nair, S., Natassia, C., Yordanova, G. et al. (2022) Alphafold protein structure database: massively expanding the structural coverage of protein-sequence space with high-accuracy models. Nucleic Acids Res. 50, D439–DD44 10.1093/nar/gkab1061 34791371 PMC8728224

[96] Atsriku, C., Hoffmann, M., Ye, Y., Kumar, G. and Surapaneni, S. (2015) Metabolism and disposition of a potent and selective JNK inhibitor [14C]tanzisertib following oral administration to rats, dogs and humans. Xenobiotica 45, 428–441 10.3109/00498254.2014.990949 25482583

[97] Ye, Y., Gaudy, A., Thomas, M., Reyes, J., Burkhardt, B., Horan, G. et al. (2022) Safety, pharmacokinetics, and pharmacodynamics of CC-90001 (BMS-986360), a c-Jun N-terminal kinase inhibitor, in phase 1 studies in healthy participants. Clin. Pharmacol. Drug Dev. 0, 1–11 10.1002/cpdd.1178 PMC10092235 36256505

[98] Beydoun, T., Deloche, C., Perino, J., Kirwan, B.A., Combette, J.M. and Behar-Cohen, F. (2015) Subconjunctival injection of XG-102, a JNK inhibitor peptide, in patients with intraocular inflammation: a safety and tolerability study. J. Ocul. Pharmacol. Ther. 31, 93–99 10.1089/jop.2013.0247 25347151

